# National health surveys are too important to depend on the mood of politics

**DOI:** 10.1590/S1518-8787.201605000SUPL2ED

**Published:** 2016-12-01

**Authors:** Aluísio J D Barros

**Affiliations:** I Programa de Pós-Graduação em Epidemiologia. Universidade Federal de Pelotas. Pelotas, RS, Brasil

This supplement presents a collection of articles based on the National Survey on Access, Use and Promotion of Rational Use of Medicines in Brazil (PNAUM). Since there is not a centralized control system for the supply of medicines in this Country, unlike many developed countries, the information obtained via population surveys are essential for evaluating the current situation of access to medicines and their use. These data are crucial for the correction of the trajectory of many policies on medicines, currently in force in Brazil, in which lots of efforts and resources are being invested.

Private expenditure on health in Brazil is high – about 53.0% of the total^2^. And among the poor, most of this expenditure (almost 80%) is aimed at the purchasing of medicines^2^. Large investments have been made in the field, from the edition of the National Medicines Policy in 1998, which aimed at ensuring the safety, effectiveness and quality of essential medicines. Several specific programs were introduced, such as the universalization of the free treatment of HIV/aids in the Country and hypertension and diabetes treatment units. More general initiatives include from the creation of generic medicines, in 1999, to the implementation of the Popular Pharmacy Program, nationwide, created in 2004, as well as other similar initiatives promoted by state governments. The programmatic bases of this process are discussed in this supplement, in the article on pharmaceutical care^1^. As a result, almost 80% of users have access to free medicines for hypertension and diabetes and about 50% of those who have chronic diseases have free access to all the medicines that they need^3^.

Surveys such as PNAUM are essential in the process of monitoring the progress of health in the Country, both regarding access to interventions (such as contraceptives, prenatal and delivery care, vaccines, medicines for chronic diseases), and the outcomes (prevalence of diseases and risk factors such as obesity). Brazil needs this information so that governments and administrations account for their actions in the field and the existing policies may be evaluated and corrected.

PNAUM began to be discussed in 2009 and took off in 2013^1^. The results are being disclosed in 2016. The survey cycle is really long and, therefore, regularity in investment and planning is essential. Political instability and lack of priority for data collection may lead to delays or abandoning of essential researches. In the case of PNAUM, the period between the beginning of the planning and beginning of implementation (four years) could have been shorter.

Other cases are just as or more severe. The Consumer Expenditure Survey (POF), carried out by the Brazilian Institute of Geography and Statistics (IBGE), had its last two rounds held in 2002-2003 and 2008-2009. Especially this last one has been intensively used for research in health. The frequency of every five or six years maintained, POF should have been held in 2013 or 2014, what did not happen. POF is scheduled for 2017, but the current political and economic crisis may derail the schedule. Even more closely related with the field of health are the national demography and health surveys (PNDS), held in 1986, 1996 and 2006. The periodicity of 10 years leaves much to be desired – five years would be better; three years, the ideal. Still, a PNDS should be held in 2016. The constant changes of ministers and instability in funding make this almost impossible. In the most optimistic scenario, a new PNDS will be held in 2017. These surveys are too important to be at the mercy of political matters. It is imperative that medium- and long-term plans are established for the surveys in the fields of health and economy, with guaranteed funding, so that the information flow is not interrupted.

At the other end of this process are scientific journals, which are responsible for disseminating the information and knowledge obtained to the academic community and society. RSP has been assuming a leading role in the country in scientific disclosing in the field of public health, with its regular articles and supplements. RSP innovated in the Country when establishing bilingual publishing of all its articles submitted in Portuguese or Spanish, seeking an effective internationalization, with the availability of quality articles accessible to all and in English. But the advancement of internationalization depends on other aspects, such as agile on-line systems of submission of articles in English and agility in the processing of the articles – from the distribution to reviewers until the final answer to authors. All of this depends on regular funding and professionalization of the journals. Scientific publication has become a big business. Meanwhile, the Brazilian journals work in isolation, and, with rare exceptions, without enough funding from development agencies, nor contribution through collection, since the majority of researchers do not have the resources to pay for publication. Investment in scientific journals can be done indirectly by development agencies, through defrayal of the publication of articles derived from projects funded by them in the journals of choice of the researchers. That is how the major funders work, such as Wellcome Trust and Bill & Melinda Gates Foundation. In Brazil, surveys are still (scarcely) funded, but not the dissemination of their results.

This supplement is an example of dissemination and survey financed by the Department of Science and Technology (DECIT) of the Ministry of Health. The investment will certainly have great impact on the Country’s policies, as well as on research in the field of pharmaceutical care. We hope that in the wake of this collection, many other articles arise, maximizing the use of the data collected.
